# Prediction of Red Blood Cell Demand for Pediatric Patients Using a Time-Series Model: A Single-Center Study in China

**DOI:** 10.3389/fmed.2022.706284

**Published:** 2022-05-19

**Authors:** Kai Guo, Shanshan Song, Lijuan Qiu, Xiaohuan Wang, Shuxuan Ma

**Affiliations:** Department of Transfusion Medicine, Beijing Children's Hospital, Capital Medical University, National Center for Children's Health, Beijing, China

**Keywords:** time-series analysis, ARIMA model, prediction, red blood cell, pediatric patients

## Abstract

**Background:**

Red blood cells (RBCs) are an essential factor to consider for modern medicine, but planning the future collection of RBCs and supply efforts for coping with fluctuating demands is still a major challenge.

**Objectives:**

This study aimed to explore the feasibility of the time-series model in predicting the clinical demand of RBCs for pediatric patients each month.

**Methods:**

Our study collected clinical RBC transfusion data from years 2014 to 2019 in the National Center for Children's Health (Beijing) in China, with the goal of constructing a time-series, autoregressive integrated moving average (ARIMA) model by fitting the monthly usage of RBCs from 2014 to 2018. Furthermore, the optimal model was used to forecast the monthly usage of RBCs in 2019, and we subsequently compared the data with actual values to verify the validity of the model.

**Results:**

The seasonal multiplicative model SARIMA (0, 1, 1) (1, 1, 0)_12_ (normalized BIC = 8.740, *R*^2^ = 0.730) was the best prediction model and could better fit and predict the monthly usage of RBCs for pediatric patients in this medical center in 2019. The model residual sequence was white noise (Ljung-Box Q_(18)_ = 15.127, *P* > 0.05), and its autocorrelation function (ACF) and partial autocorrelation function (PACF) coefficients also fell within the 95% confidence intervals (CIs). The parameter test results were statistically significant (*P* < 0.05). 91.67% of the actual values were within the 95% CIs of the forecasted values of the model, and the average relative error of the forecasted and actual values was 6.44%, within 10%.

**Conclusions:**

The SARIMA model can simulate the changing trend in monthly usage of RBCs of pediatric patients in a time-series aspect, which represents a short-term prediction model with high accuracy. The continuously revised SARIMA model may better serve the clinical environments and aid with planning for RBC demand. A clinical study including more data on blood use should be conducted in the future to confirm these results.

## Introduction

Red blood cells (RBCs) are an indispensable aspect of clinical blood transfusion considerations, with transfusions being vital for supplementing a patient's blood oxygen level, especially in the care of cases critically ill patients, elective and emergency surgery, major trauma, hemorrhage, cancer care, and for supporting patients with congenital or acquired anemia (1, 2). However, RBCs have an effective storage time outside the body (3). Under the current technical conditions in China, RBCs can generally be stored for up to 35 days (when RBCs are combined with an additive solution, which is usually citrate phosphate dextrose adenine, CPDA-1). Due to the limitations of the storage period, the accumulation of too much inventory may cause blood waste, and insufficient inventory may delay treatment time and even endanger the lives of patients. Currently, the principle of the supply management of ready-to-use blood products has been developed to minimize blood wastage. Moreover, blood availability may need to be increased again to meet the demands of aging populations in the future (4). Growing blood products in large quantities to match blood demand can result in many challenges.

Blood transfusion is also crucial supportive care for pediatric patients. According to our previous investigation, the prevalence of transfusions in pediatric inpatient hospitalizations was 6.19% in the mainland of China (5). Moreover, it is currently known that children are not similar to small adults (6); as children have a different biochemical profile (7). As far as children are concerned, their weights are less than that of adults, and diseases can rapidly progress; furthermore, their blood usage specifications are represented mainly by small packages, which are flexible and changeable. Therefore, blood transfusion strategies for neonates and children cannot follow principles similar to those for adults (8–12). Additionally, the number of visits for pediatric patients, especially children undergoing surgery, is related to the frequencies of winter and summer vacations in China. There is also substantial variability in the proportion of transfused children, and several similar yet unique, non-laboratory predictors of transfusion have been identified in these children (13).

Generally, the calling of donors and the scheduling of blood collection and supply must be coordinated to match the clinical demand for patients, especially in regard to the manufacturing and supply of RBCs to hospitals or medical centers. To ensure a balance between the blood demand of the hospitals and the blood supply of the Red Cross Blood Center or blood bank, as well as avoiding the phenomenon that the hospital blood inventory may be in a shortfall or a state of oversupply, the hospital or medical center must predict its blood consumption so that the foresight, rational allocation of blood resources, and coordination of clinical needs of the blood center or blood bank can make clinical blood management more reasonable and scientifically sound.

Currently, some tentative approaches to predict RBC demand in simple deterministic models have been performed by utilizing demographic for age distribution, the age-specific prevalence of diseases, donation frequency of donors, donor recruitment rates, numbers of donations, RBC transfusion data, and/or the blood requirements according to the indications for different diseases, etc. (14–19). Nevertheless, such models have not been able to accurately predict the changes in clinical transfusion practices (15, 20, 21). Using a time-series method may be an excellent strategy to forecast the demand for RBC transfusion with high accuracy. Time-series models have been employed in a wide variety of research fields, including public health and biomedical data aspects (22), brain studies (23), drug utilization (24), gene networks (25, 26), and even traffic safety (27). Some studies have also examined the time-series prediction of the demand for RBCs (28–30). A trend model, which included time-series models with exponential smoothing and generalized additive regression models, was constructed to estimate the demand for RBC transfusion by 2035 in Switzerland (31). This prediction method may be developed to provide reliable and precise prediction effects and improve the efficiency of blood services, as well as the sufficiency of the blood supply.

Therefore, we used a time-series, autoregressive (AR) integrated moving average (MA) (ARIMA) model to estimate the number of RBCs for pediatric patients in the children's hospital. The ARIMA model is one of the most important methods of time-series analysis. Specifically, it can combine the advantages of continuous models and seasonal models, and it is also more suitable for the establishment of clinical blood data series models that involve more complex factors. It has been applied to many fields (32–36). This study aimed to establish an ARIMA model by selecting appropriate parameters for the prediction of the clinical needs of RBCs in children, as well as to evaluate the feasibility of the model and to provide a basis for the rational planning of blood use in the future, with the ultimate goal of enacting a clinical transition from empirical blood preparation to scientific blood preparation.

## Materials and Methods

### Study Population and Source of Data

The study population was composed of pediatric patients aged 0 to 18 years from Beijing Children's Hospital, Capital Medical University, National Center for Children's Health of China. Beijing Children's Hospital is a comprehensive 3A pediatric hospital with 970 beds and an average of 3 million visits to outpatients, more than 70 000 total inpatient admissions, over 23,000 operations per year (37). These retrospective transfusion data included monthly aggregates of RBC units that were used over a period of 6 years from January 2014 through December 2019 for pediatric patients, and these data were obtained from the Blood Transfusion Manage Information System (BTMIS) in this medical center. BTMIS is an electronic database that is used to store these data.

After careful inspection of the actual clinical data of RBC usage, it became clear that there were considerable fluctuations from day to day, weekday to weekend, and week to week. Therefore, we used aggregated data from month to month in this study rather than daily or weekly data. According to our dataset, the monthly usage of RBCs can change within 30% (mostly within 20%) from month to month.

### RBC Product Unit

One unit of RBC (U) was obtained from 200 ml of whole blood, and usually, CPDA-1 solution was added to the RBC unit to a final volume of 160 ± 10% mL and hemoglobin (Hb) ≥ 20 g (RBC in additive solution) or a final volume of 150 ± 10% mL, Hb ≥ 18 g, and leukocytes ≤ 2.5 × 10^6^. The latter RBC units were RBCs in additive solution leukocytes reduced, after which they were stored between 4 and 6°C for up to 35 days. Other RBC products included washed RBC (total volume = 125 ± 12.5 mL, Hb ≥ 18 g), deglycerolized RBC (total volume = 200 ± 20 mL, Hb ≥ 16 g, and glycerin residue ≤ 10 g/L) and irradiated RBC components.

In this prediction study, all types of RBC products were uniformly adjusted to RBCs for ease of calculation. RBC components were generally provided by the Beijing Red Cross Blood Center in accordance with Chinese national quality standards in our medical center.

### Model Construction

A time-series analysis explores the development processes and trends reflected by a set of values arranged in a specific time interval. Time-series analyses assume that the observations follow a regular pattern over time, and these observations are often contaminated by random noise (28). Therefore, identifying the hidden pattern is difficult. However, unknown observations can be predicted if the pattern is identified.

The ARIMA model is a time-series analysis model that can capture varying time characteristics of a set of observations through time (t); it can identify the dependence and autocorrelation between the data and then establish a model to predict its development trend. The ARIMA model considers each observation related to the preceding one, allowing analysis at a specific time point relative to the previous dependent, time-lagged ones via an AR and/or a MA process (28, 38). The model specification can be mainly based on the following formulations (28, 38):


(1) $$\begin{array}{l} AR_{\left(\text{p}\right)}:Y_{\text{t}}=\varepsilon +\alpha_{1}Y_{\text{t}-1}+\alpha_{2}Y_{\text{t}-2}+\ldots +\alpha_{\text{p}}Y_{\text{t}-\text{p}}+\beta_{\text{t}} \end{array}$$

For the above equation, ε: constant; β_t_: white noise process; p: order. That is, each observation is composed of a linear function of the preceding p observations and a random shock (β_t_) occurring at time t.


(2) $$\begin{array}{l} MA_{\left(\text{q}\right)}:Y_{\text{t}}=\mu +\beta_{\text{t}}+\alpha_{1}\beta_{\text{t}-1}+\alpha_{2}\beta_{\text{t}-2}+\ldots +\alpha_{\text{q}}\beta_{\text{t}-\text{q}} \end{array}$$

Where; μ: constant; q: order. Each observation is also made up of a random shock and a linear function of the q prior random shocks. The recursive calculation is used to estimate the coefficients (α) and the constants (ε and μ).

A combined ARMA (p, q) process can be derived from the two previous equations. The ARIMA (p, d, q) model combines non-parametric differencing and integration with a parametric ARMA process, where d is the number of differencing operations. The “I” in the ARIMA acronym represents this time-series integration process.

Microsoft Excel Version 2016 and IBM SPSS 26.0 version software (IBM Corp., Armonk, NY., USA) was used to establish a monthly clinical RBC consumption database of pediatric patients in our medical center. Additionally, we used relevant statistical modules for data processing and analysis, and we established an ARIMA model through the following four steps.

### Sequence Smoothing

The ARIMA model is based on the assumption that the time series is stationary, which is rarely the case in practice. If time-series points are non-stationary, which means that there is an apparent trend, irregular variation, or seasonality in the time series, the original data need to be preprocessed, such as first-order or high-order differencing, separations of deterministic components, zero averaging, and the Box-Cox transform, which is a configurable data transform method, etc., to produce the stationary time-series. Generally, the differencing (d) process is needed for the integrated process to make the non-stationary sequence stable.

Summarily, the time series used for analysis and modeling must meet the requirements of stationarity and randomness; specifically, the first-order and second-order parameters of the series do not change with time (t). After obtaining a stationarity sequence, an ARIMA model can then be established. The time-series graph is the corresponding series graph drawn after converting the original data.

### Outlier Detection

The point was considered to be an outlier when the value at a certain time (t) exceeded a certain range, and it was eliminated. The number of outliers was zero in this study.

### Model Identification

Through the use of autocorrelation plots, including the autocorrelation function (ACF) and partial autocorrelation function (partial ACF, or PACF), we explored the approximate parameter order of the model. Specifically, defining an ARIMA (p, d, q) model requires finding the times (d) of the differencing that are necessary to make a non-stationary sequence stationary and the order of the AR_(p)_ and/or MA_(q)_ parameters. Generally, p and q values can be preliminarily determined with the help of the ACF and the PACF plots. For example, if the ACF graph is truncated of order q, the MA coefficient is q; if the PACF graph is truncated of order p, the AR coefficient is p.

### Model Parameter Estimation and Testing

After the model was established, parameter estimation and hypothesis testing were required to determine whether the model was suitable. Basically, Melard's algorithm is used to estimate the parameters in the ARIMA model when there are no missing observations. The details of this algorithm are described elsewhere (39–41). A Kalman filtering algorithm (42) can be used for an ARIMA model with missing data in the time series. The white noise test is one of the most commonly used methods for a diagnostic test of the ARIMA model, and its test is represented by Ljung-Box Q statistic (43). Simultaneously, the ARIMA model can be evaluated by observing whether ACF and PACF coefficients of the residual sequence fell within the 95% confidence intervals (CIs). Finally, the best-fitting model can be selected according to the normalized Bayesian Information Criterion (BIC) and *R*^2^. Smaller normalized BIC values and bigger *R*^2^ indicate better model fitting.

Briefly, we first find the order of models. The parameters are then estimated, and the models are chosen if they are statistically significant. If more than one model is accessible based on significant parameters and residual tests, the best model can be chosen based on BIC and *R*^2^.

### Model Prediction

The optimal model was used to forecast RBC consumption and the corresponding 95% CI of pediatric patients in our hospital in 2019. The forecasting effect of the model was evaluated through the use of comparisons with actual data.

### Statistical Analysis

In this study, α = 0.05 was used as the test level, and IBM SPSS 26.0 version software (IBM Corp., Armonk, NY., USA) was used to analyze the data.

## Results

### Time Series Characteristics of Monthly RBC Usage

**Figure 1:**
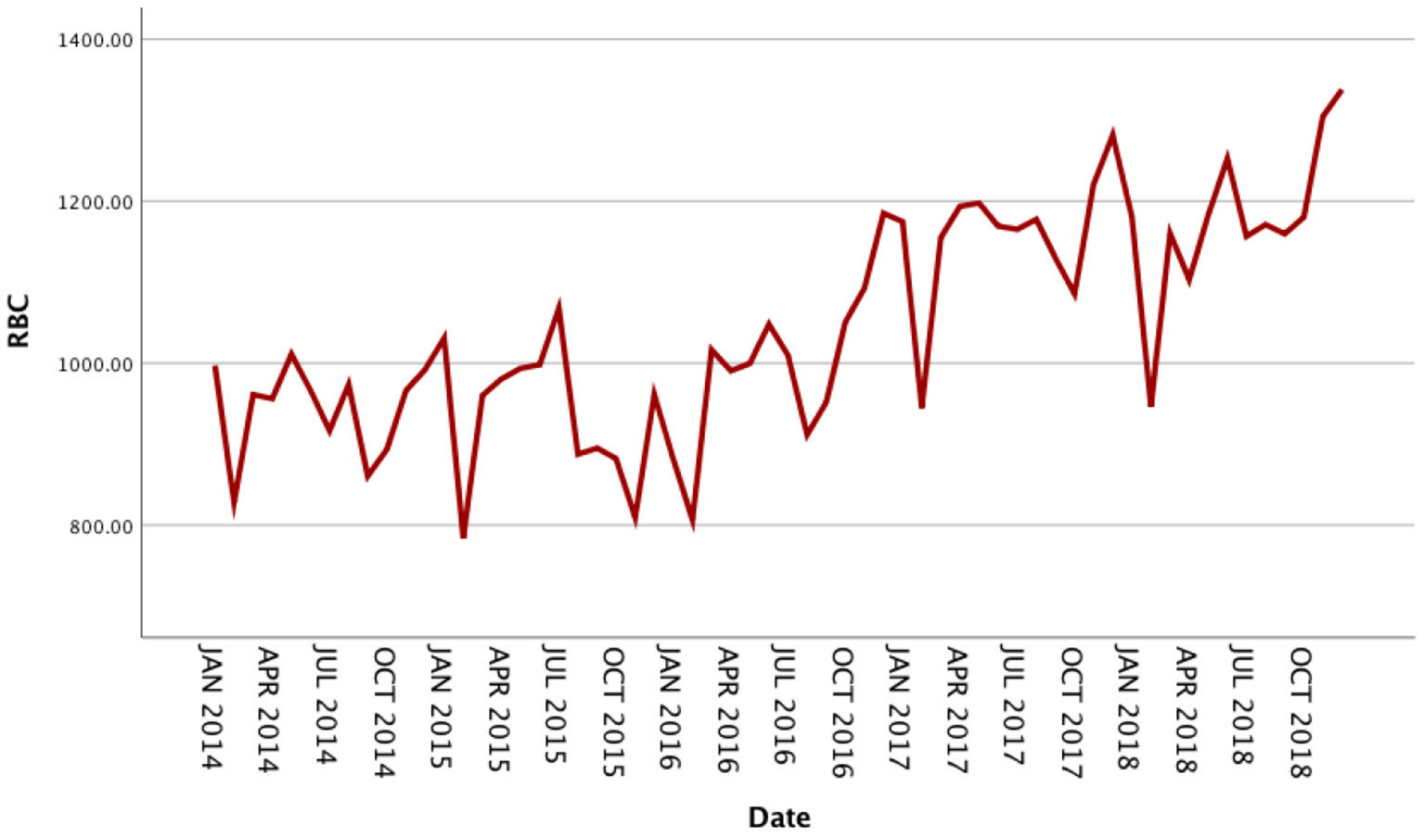
Original sequence diagram of monthly usage of red blood cells from 2014 to 2018.

**Figure 2:**
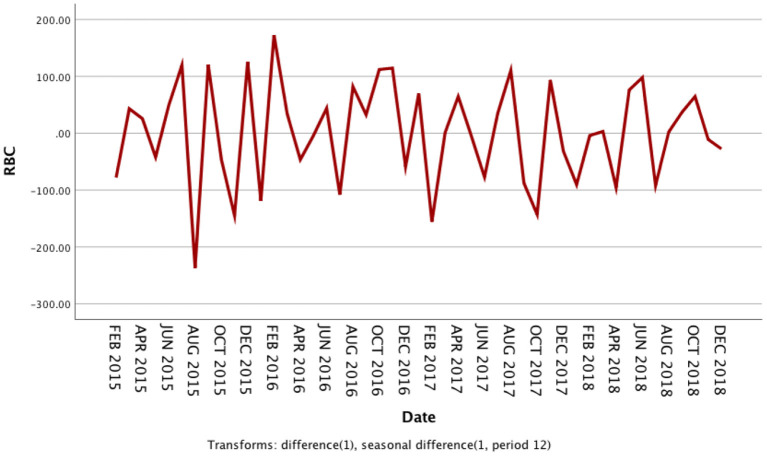
Sequence diagram after first-order non-seasonal differencing and seasonal differencing in monthly usage of red blood cells from 2014 to 2018.

The usage of RBCs in pediatric patients in our medical center from January 2014 to December 2018 is shown in Supplementary Table 1. The RBC consumption of pediatric patients demonstrated a specific seasonal cycle, and the annual RBC consumption showed a slight upward trend. The original sequence diagram of monthly RBC consumption is shown in Figure 1. The rising trend and seasonal periodicity indicated that the sequence was non-stationary. To eliminate the influence of the original sequence's trend and seasonal periodicity, non-seasonal and seasonal differencing processing was performed. After processing, each value in the sequence approximately fluctuated around the zero mean value; that is, the fluctuation was a relatively stationary sequence (Figure 2).

### Model Identification and Parameter Estimation

**Figure 3:**
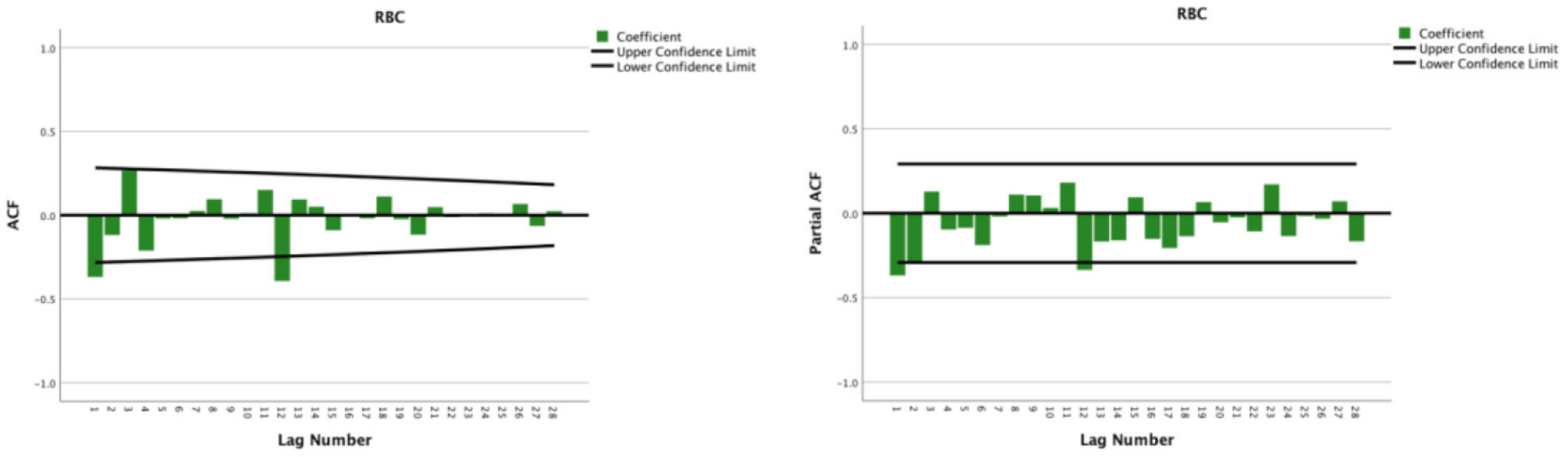
Autocorrelation function and partial autocorrelation plots after the first-order non-seasonal differencing and seasonal differencing of monthly usage of red blood cells from 2014 to 2018.

The seasonal multiplicative ARIMA model SARIMA (p, d, q) (P, D, Q)s, which is one of the models from which seasonal effects can be extracted and has certain representativeness and universality, was chosen to predict RBC demand because the original sequence of RBC usage had seasonal periodicity. The “s” is the seasonality or period of the model. The original sequence of RBCs represents 12 months as a cycle; s = 12. The p, d, q, and P, D, and Q are the continuous and seasonal ARIMA order values, respectively, and are the number of time points contained in a seasonal cycle. The first-order non-seasonal differencing and the first-order seasonal differencing were determined. After the first-order differencing, the mean value of the series fluctuated around the zero mean value, and there were no apparent changes in the trends (Figure 2), thus indicating that the series had stabilized, which met the requirements of the ARIMA model and verified that it could be used for the model identification process; therefore, d = 1 and D = 1. Furtherly, the ACF and PACF plots were drawn according to the time series after the differencing, as shown in Figure 3. The ACF and PACF were significantly non-zero at lags 1 and 12 order; therefore, assuming q was taken at 1 or 12, p was also accepted at 1 or 12, and try the case where q and p were taken at 0 for the model to be more fully considered. Generally, the seasonal autoregressive order P and the seasonal moving average order Q are challenging to determine. Nevertheless, P and Q do typically not exceed 2, i.e., 0, 1, or 2.

### Model Statistical Test and Assessment on the Best-Fitting Model

**Figure 4:**
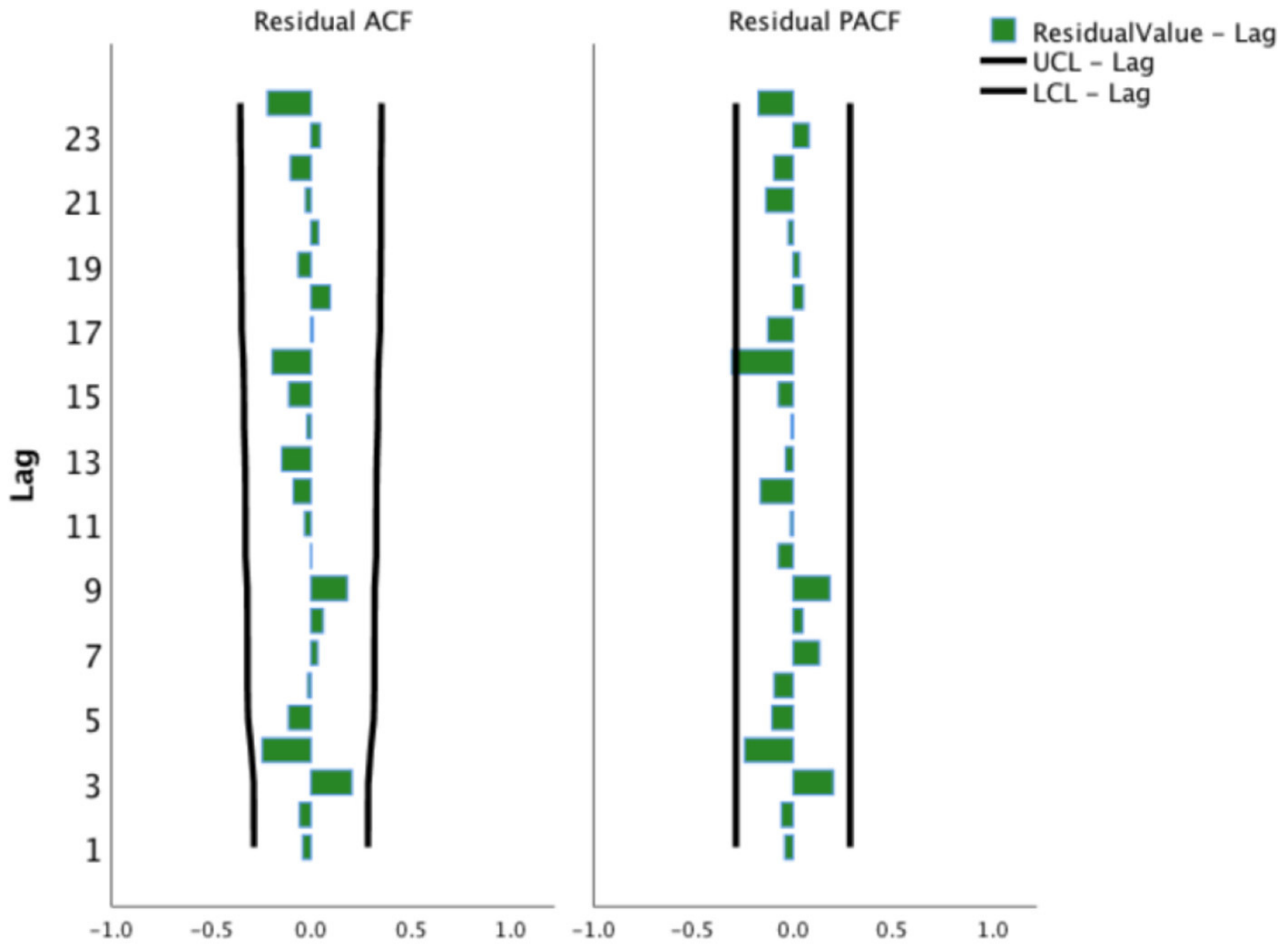
Autocorrelation function and partial autocorrelation function plots of the residual sequence of the SARIMA (0, 1, 1) (1, 1, 0)_12_ model. UCL, Upper Confidence Limit; LCL, Lower Confidence Limit.

**Figure 5:**
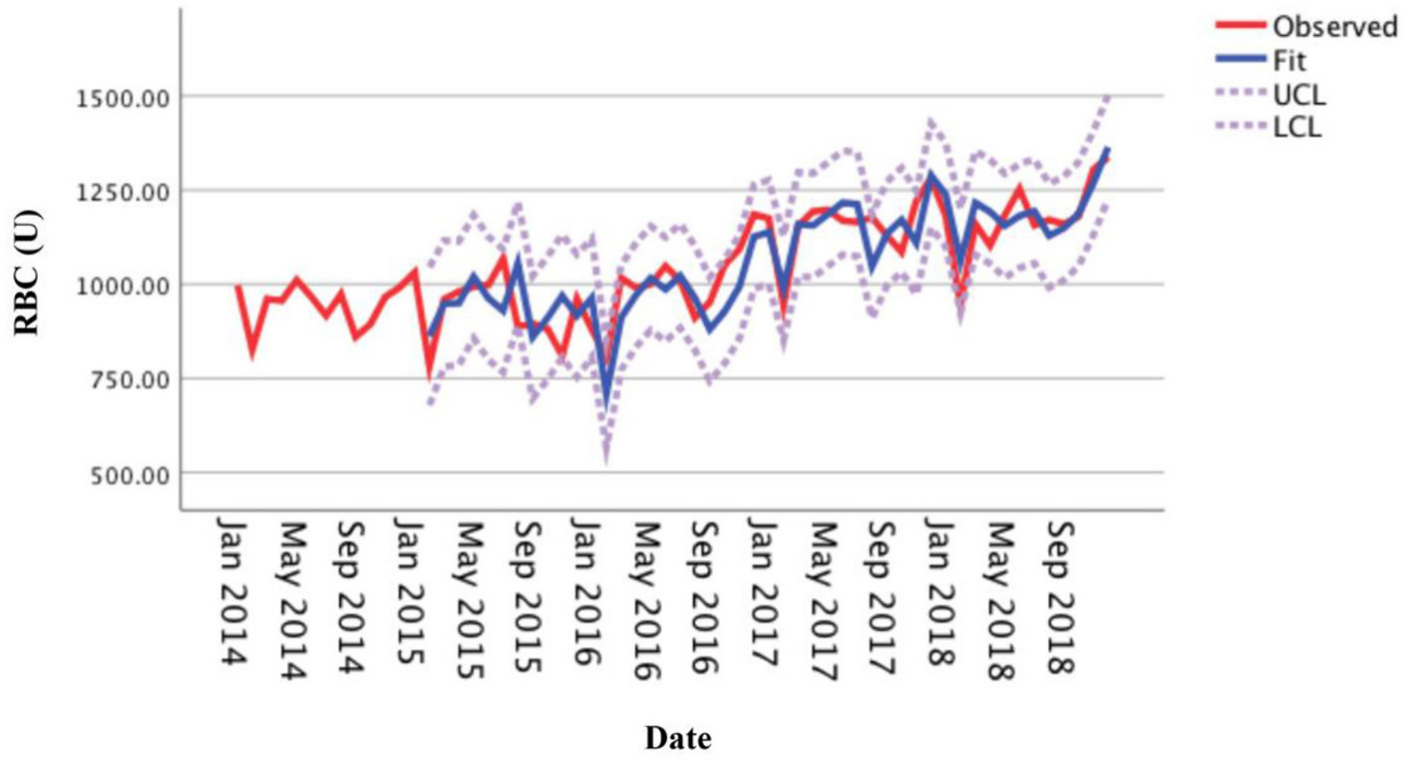
Fitting effect of the SARIMA (0, 1, 1) (1, 1, 0)_12_ model for the monthly usage of red blood cells.

This study adjusted the parameters to try different SARIMA models with the help of the ACF and the PACF plots and performed parameter estimation and statistical tests. The SARIMA (0, 1, 1) (1, 1, 0)_12_ model (did not contain a constant term) was the best model among these models according to significant parameters and residual tests, and normalized BIC and *R*^2^. The results of the parameter test were statistically significant (Table 1, *P* < 0.05). The ACF and PACF coefficients of the residual sequence also fell within the 95% CIs, which indicated the error term was random (Figure 4). The white noise test was performed on the residual sequence, with Ljung-Box Q_(18)_ = 15.127, *P* = 0.515. The residual series can be considered white noise, and the model had fully extracted information. Normalized BIC = 8.740, *R*^2^ = 0.730. The prediction fitting effect is shown in Figure 5. The fitting curve was basically consistent with the actual observation curve.

**Table 1:** Parameter estimation and statistical test of the SARIMA (0, 1, 1) (1, 1, 0)_12_ model.

**Parameter**		**β**	**Standard error**	***t-*value**	***P-*value**
No transformation	Differencing	1			
	MA	0.509	0.130	3.906	<0.001
	AR, seasonal	−0.535	0.129	−4.138	<0.001
	Seasonal differencing	1			

### Actual Forecasting Analysis

**Figure 6:**
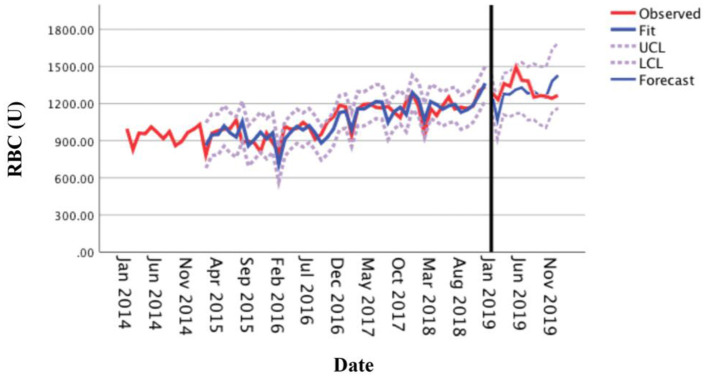
Comparison between the forecasted and actual values of the monthly usage of red blood cells in 2019.

The optimal forecasting model SARIMA (0, 1, 1) (1, 1, 0)_12_ established by monthly RBC consumption of the medical center from 2014 to 2018 was used to forecast the monthly RBC consumption in 2019. The results showed that the actual RBC consumption (observed curve) in each month of 2019 was within the 95% CI of the forecasted value (Figure 6), with 91.67% accuracy, and the average relative error of the forecasted and actual values was 6.44% (within 10%), indicating that the model has an excellent forecasting fit.

## Discussion

Simple linear models were previously used to forecast medium-term blood demand (16, 44). In general, these methods have predicted an increasing blood demand based on aging demographics (45), while there may be a potential shortage from 18- to 24-year-olds blood donations (46). Although these methods have provided some significant and concrete evidence for planning blood supply demands, they could not satisfactorily predict the changing trends in blood demand (21, 47), and short-term planning has relied on time-series methods (30).

The ARIMA model is one of the most widely used time series models. It can screen out the optimal forecast model through the repeated identification, modification, and diagnosis of the model. Additionally, it has strong applicability and high accuracy, and it can comprehensively extract trend information and periodic information in a time series to predict future changes. The seasonal variation index cannot be used universally for different medical institutions in the medical and health fields. It is necessary to obtain a seasonal index suitable for the characteristics of the medical institution based on that specific institution's observation data. Therefore, it is difficult to predict with the use of traditional regressions. The SARIMA model is a combination of multiple time series models and is suitable for forecasting clinical RBC demand related to seasonal cycles.

This study used the SARIMA model to statistically analyze and model the monthly RBC time series data of pediatric patients in our medical center from January 2014 to December 2018 on a month-by-month basis. Subsequently, we used the optimal model SARIMA (0, 1, 1) (1, 1, 0)_12_ to forecast the time series of monthly RBC usage in 2019. The results showed that the 91.67% actual values were within the 95% CI of the forecasted value, and the average relative error of the forecasted and actual values within 10% (6.44%), thus indicating that the short-term prediction accuracy was high and had an excellent fitting effect. According to Pereira A's study (28), the RBC transfusion series was also well fitted by the SARIMA model. The model produced monthly forecasted values within ±10 % of the actual demand for RBCs in 79% of the months over a 1-year horizon. One study predicted the monthly type A, B, O, and AB RBC usage and total usage using the SARIMA method based on the data of the Central Blood Station of Wanzhou, China; the predicted values were compared to the actual values, and the mean relative errors were 9.2, 7.5, 4.9, 10.8, and 4.3%, respectively (48). Similar to our findings. The SARIMA model may be generalized in the forecasting of clinical RBC consumption in medical institutions and can provide a basis for the scientific prediction of clinical blood demand and reasonable blood preparation.

Currently, other models have also been used to predict total blood/blood products demand. However, these models need to take account into numerous variables, such as a Poisson generalized estimating equations model involved in transfusion patients with demographics (age, sex, weight), years of transfusion and history of splenectomy (44), and an eXtreme Gradient Boosting model involved in RBC transfusion statistics, the time factor, holiday factor, mean transfusion of departments, the purpose of applying transfusion, application date and quantity requested (49). It would be beneficial to assess these models' applicability and respective advantages in predicting total blood and blood product demand through a systematic review with sensitivity analysis. Additionally, emerging machine learning models for intelligent prediction of RBC transfusion seem to be beneficial to disease-specific patients, such as trauma patients (50), preoperative patients (51), the patients undergoing mitral valve surgery (52), and the patients during or after liver transplantation surgery (53). These methods combined with time-series models may help us predict short-, medium-, or/and long-term RBC demand more accurately and conveniently in the future.

### Limitations

It must be noted that several limitations need to be mentioned in this study. First, the results are promising even though the data set is small; our study only provided a single-institution retrospective observational report; whether the forecasting could be hold in other hospitals/institutes is unknown since the data is only from one hospital. Second, the SARIMA model is based on the statistical analysis of past data to establish a model and is only suitable for short-term forecasts. Due to the fact that the model highlights the time series and does not take into account the influence of other factors, the SARIMA model may have a forecast error defect. If there were major changes, such as the outbreak of coronavirus pneumonia at the end of 2019, it would have an extreme impact on the forecasting of RBC demand for 2020 (data not shown). Moreover, the various conditions established by the SARIMA model can only remain stable for a certain period of time. Therefore, medical institutions should continuously modify or refit the model according to the actual situation to improve the prediction accuracy and ensure the fitting effect to provide a basis for the clinical formulation of blood use plans in a timely and accurate manner.

## Conclusions

In conclusion, in this study, time-series analysis methods were used to develop a forecasting model SARIMA (0, 1, 1) (1, 1, 0)_12_ of RBC demand month-to-month, which was expedient and had an excellent performance and provided a solid quantitative basement on which to make the future RBC demand plan. The continuously revised SARIMA model may better serve the clinical environments and aid with planning for RBC demand. It may be beneficial in these eras of limited resources and narrowing blood supply and transfusion margins.

## Data Availability Statement

The original contributions presented in the study are included in the article/Supplementary Material, further inquiries can be directed to the corresponding author.

## Ethics Statement

The study involving medical data was reviewed and approved by the Ethics Committee of Beijing Children's Hospital, Capital Medical University.

## Author Contributions

KG: conception and design. KG and SM: administrative support and data analysis and interpretation. KG, SS, LQ, and XW: collection and assembly of data. All authors: manuscript writing and final approval of the manuscript.

## Conflict of Interest

The authors declare that the research was conducted in the absence of any commercial or financial relationships that could be construed as a potential conflict of interest.

## Publisher's Note

All claims expressed in this article are solely those of the authors and do not necessarily represent those of their affiliated organizations, or those of the publisher, the editors and the reviewers. Any product that may be evaluated in this article, or claim that may be made by its manufacturer, is not guaranteed or endorsed by the publisher.
