# Supplementary material for: Prediction of Red Blood Cell Demand for Pediatric Patients Using a Time-Series Model: A Single-Center Study in China
Source: Front Med (Lausanne). 2022 May 19;9:706284. doi: 10.3389/fmed.2022.706284 (PMC9162489; doi:10.3389/fmed.2022.706284)
Supplement: Supplementary file 1 [file Table_1.DOCX]

**TABLE S1** | Monthly consumption of red blood cells in our medical center from 2014-2018 (U).

| **Year** | **2014** | **2015** | **2016** | **2017** | **2018** |
| --- | --- | --- | --- | --- | --- |
| **Month** |  |  |  |  |  |
| 1 | 997 | 1031 | 881 | 1174.5 | 1180.5 |
| 2 | 828 | 784 | 806.5 | 944.5 | 946.5 |
| 3 | 961 | 960 | 1016.5 | 1155 | 1160 |
| 4 | 956 | 980.5 | 990.5 | 1193.5 | 1103 |
| 5 | 1011 | 993.5 | 999.5 | 1197.5 | 1183 |
| 6 | 966.5 | 998 | 1048 | 1169 | 1252.5 |
| 7 | 916.5 | 1067.5 | 1009.5 | 1165 | 1156.5 |
| 8 | 973.5 | 887.5 | 911.5 | 1177.5 | 1171 |
| 9 | 860.5 | 895 | 951.5 | 1129.5 | 1159.5 |
| 10 | 893.5 | 882 | 1050.5 | 1085.5 | 1180 |
| 11 | 966 | 809.5 | 1092.5 | 1221 | 1304.5 |
| 12 | 992 | 961 | 1185 | 1281.5 | 1337.5 |
